# Global Trends in Mortality and Burden of Stroke Attributable to Lead Exposure From 1990 to 2019

**DOI:** 10.3389/fcvm.2022.870747

**Published:** 2022-06-23

**Authors:** Tongchao Zhang, Xiaolin Yin, Yuan Zhang, Hui Chen, Jinyu Man, Yufei Li, Jiaqi Chen, Xiaorong Yang, Ming Lu

**Affiliations:** ^1^Clinical Epidemiology Unit, Qilu Hospital of Shandong University, Jinan, China; ^2^Clinical Research Center of Shandong University, Jinan, China; ^3^Department of Epidemiology and Health Statistics, School of Public Health, Cheeloo College of Medicine, Shandong University, Jinan, China

**Keywords:** stroke, lead exposure, mortality, disability-adjusted life-years, temporal trend

## Abstract

**Background:**

Lead exposure is an important risk factor for stroke. However, the latest global spatiotemporal patterns of lead exposure-related stroke burden were unclear. In this study, we assessed this topic.

**Methods:**

The data were obtained from the Global Burden of Disease Study (2019). The estimated annual percentage change (EAPC) was estimated to evaluate the temporal trends of the age-standardized mortality and disability-adjusted life years (DALYs) rates (ASMR and ASDR) of stroke attributable to lead exposure.

**Results:**

In 2019, the numbers of global stroke deaths and DALYs attributable to lead exposure were 305.27 and 6738.78 thousand, respectively. The corresponding ASMR and ASDR were highest in males, the elderly population, low and middle-income countries, and the intracerebral hemorrhage subtype. From 1990 to 2019, the ASMR and ASDR of global stroke attributable to lead exposure decreased [ASMR: EAPC = −1.34, 95% confidence interval (CI): (−1.57, −1.10); ASDR: EAPC = −1.74, 95% CI: (−1.95, −1.52)], especially in females, the high-income countries, and the subarachnoid hemorrhage subtype.

**Conclusion:**

This study emphasizes the importance of continued implementation of lead exposure prevention strategies and improved high-efficiency treatment and stroke acute health care, especially in low and middle-income countries.

## Introduction

Stroke is a major disease that brings a heavy burden worldwide (1). Globally, stroke contributes to 6.55 million deaths and 143.23 million disability-adjusted life years (DALYs) in 2019 (2). With the improvement of treatment, the age-standardized rates of mortality and DALYs (ASMR and ASDR) have decreased during the past decades, however, the burden of stroke remains high in most regions and countries (1, 3). In 2019, stroke was still the third cause of DALYs in all ages and was the second leading cause of DALYs in the population aged 50 years old (4). Previous evidence suggests that about 90% of the strokes could be attributed to modifiable risk factors, such as smoking, physical inactivity, high body-mass index, poor diet, and air pollution (1, 5). For every dollar spent on preventing stroke and cardiovascular disease, an estimated return on investment of 10.9 dollars would be brought (6). Therefore, the implementation of effective preventive strategies might be the most effective way to reduce the burden of stroke.

Lead is a heavy metal widely applied in industry and has been recognized by the World Health Organization (WHO) as one of the major environmental pollutants causing public health problems (7). According to the latest Global Burden of Disease (GBD) data, in 2019, almost 0.90 million deaths and 21.68 million DALYs were attributed to lead exposure around the world (8). Lead can be exposed to humans through food, gasoline, paint, and polluted water, air, or soil (9), and is mainly absorbed from the respiratory or gastrointestinal tracts and accumulates in bone, blood, and soft tissues (9). Due to the cumulative effect of lead exposure, it may adversely affect multiple body systems, including respiratory, nervous, cardiovascular digestive, and kidney systems (10). Previous evidence has proved that lead exposure is an important risk factor for cardiovascular disease (including stroke) (9, 11–16), and the National Health and Nutrition Examination Survey cohort study in the US found a dose-response relationship between blood lead level and stroke mortality (17). Moreover, another study found that, from 1990 to 2014, about 34,000–99,000 deaths have been avoided due to the decline in blood lead levels (11). Lead exposure might contribute to the development of stroke by inducing inflammation (9, 18), increasing oxidative stress levels (16, 19, 20), and altering immune regulation through cytokines and immune cells (10, 21–23).

A comprehensive assessment of the stroke burden attributable to lead exposure will help to formulate effective prevention strategies and reduce the stroke burden. However, few studies evaluated the burden and changing trend of stroke attributable to lead exposure. In this study, using the latest database of the GBD study (2019), we comprehensively evaluated the global burden and changing trends of mortality and DALYs of stroke attributable to lead exposure at global, regional, and national levels. Our results can provide an important basis for the prevention of stroke.

## Materials and Methods

### Data Collection

The detailed data processing and modeling methods of the GBD study (2019) have been fully reported in previous studies (2, 4), and the codes used to assess stroke burden in the GBD database can be obtained from the following website: http://ghdx.healthdata.org/gbd-2019/code. The annual information on the global stroke burden attributable to lead exposure was obtained from the Institute for Health Metrics and Evaluation (query tool)^1^ based on the following screening criteria: first, the locations were global, regions, and countries, the year was choose from 1990 to 2019, and the risk was “lead exposure.” Second, the causes were “stroke,” “ischemic stroke (IS),” “intracerebral hemorrhage (ICH),” and “subarachnoid hemorrhage (SAH).” Third, the measures were “Deaths” and “DALYs.”

The GBD (2019) contained 204 countries or territories, these countries or territories were geographically classified into 21 regions and nested within 7 super regions (24). Meanwhile, the 204 countries or territories were also grouped into 5 regions (low, low-middle, middle, high-middle, and high) based on the socio-demographic index (SDI), a comprehensive indicator estimated according to fertility rate, years of education, and income (25).

### Definition and Evaluation of Stroke and Lead Exposure

In the GBD study, stroke was defined based on the standards of WHO and was pathologically divided into IS, ICH, and SAH subtypes (2). There were two definitions of lead exposure: acute lead exposure and chronic lead exposure (26). Acute lead exposure was measured in micrograms of lead per deciliter of blood (μg/dl) and associated with IQ loss in children; while chronic lead exposure was measured in micrograms of lead per gram of bone (μg/g) and associated with increased systolic blood pressure and cardiovascular disease (26).

Verbal autopsy and vital registration data were used to estimate the stroke burden in the GBD study (4). Stroke deaths were modeled by using the standard Cause of Death Ensemble modeling method after adjusting for the Covariates (4). The blood lead exposure data were extracted from the research reports and blood lead surveys (552 studies from 84 countries), while the bone lead exposure data were evaluated by estimating a cumulative blood lead index for cohorts using estimated blood lead exposure over their lifetime (26). The comparative risk assessment framework was conducted to evaluate the population attributable fractions of lead exposure for stroke burden (2, 27, 28). For detail, the relative risk was estimated and the exposure levels were estimated using the spatiotemporal Gaussian process regression, Bayesian meta-regression method, and other methods; then theoretical minimum risk exposure level was defined and the population attributable fractions and attributable burden were calculated (2, 27, 28).

### Statistical Analysis

ASMR and ASDR were estimated by first calculating the sum of the products of the age-specific rates and the same age group number (or weight) of the standard population GBD (2019) (29) world population age distribution, and then dividing by the sum of the standard population number (or weight) (30, 31). DALYs were calculated by adding the years lived with a disability to the years of life lost (31). We described the numbers and age-standardized rates of stroke burden attributable to lead exposure and their 95% uncertainty intervals (UIs) by year, sex, pathological subtype, region, and country (24, 25). 95% UIs were generated based on the 2.5th and 97.5th centiles of 1,000 random draws of the uncertainty distribution (24, 25).

The indicator of estimated annual percentage change (EAPC) was used to evaluate the temporal trend from 1990 to 2019. A regression model was performed to fit the natural logarithm of the age-standardized rate [ln (age-standardized rate) = α + β*(calendar year) + ε], then EAPC and its 95% confidence interval (CI) was calculated [100 × (exp(β)−1)] (24, 25, 28, 30, 31). When the upper boundary of 95%CI of EAPC was less than 0, it was considered that the changing trend was decreasing; while when the lower boundary of 95%CI of EAPC was more than 0, it was considered that the changing trend was increasing; otherwise, it was considered that the changing trend remained stable during this period (27, 28, 30, 31).

Moreover, we also evaluated the associations between EAPC, lead exposure-related stroke burdens, and SDI using the Spearman rank test (27, 28). All statistical analyses and visualization of results were performed using the R program (Version 4.0.3; R Foundation for Statistical Computing, Vienna, Austria), and the two-tailed *P-*value < 0.05 was considered statistically significant.

## Results

### Global Burden of Stroke Attributable to Lead Exposure in 2019

The number of stroke deaths and DALYs attributed to lead exposure in 2019 were 305.27 thousand (95% UI: 182.80, 435.67) and 6738.78 thousand (95% UI: 3912.20, 9815.60) (Tables 1, 2), accounting for 4.66 and 4.70% of the total number of stroke, respectively. The global ASMR and ASDR in 2019 were 3.85 per 100,000 (95% UI: 2.30, 5.50) and 81.97 per 100,000 (95% UI: 47.86, 119.11), respectively (Tables 1, 2).

**TABLE 1:** Stroke deaths and age-standardized mortality rate attributable to lead exposure in 1990 and 2019, and its estimated annual percentage change from 1990 to 2019.

Characteristics	1990		2019		1990–2019
	Deaths No. × 10^3^ (95% UI)	ASMR per 100,000 No. (95% UI)	Deaths No. × 10^3^ (95% UI)	ASMR per 100,000 No. (95% UI)	EAPC in ASMR No. (95% CI)
Overall	207.41 (124.08, 304.80)	5.53 (3.28, 8.22)	305.27 (182.80, 435.67)	3.85 (2.30, 5.50)	−1.34 (−1.57, −1.10)
**Sex**					
Males	128.24 (81.43, 181.88)	7.64 (4.87, 10.88)	189.76 (118.97, 265.43)	5.36 (3.39, 7.48)	−1.29 (−1.54, −1.03)
Females	79.18 (40.70, 124.23)	3.88 (1.97, 6.13)	115.51 (62.97, 177.15)	2.63 (1.43, 4.03)	−1.46 (−1.67, −1.25)
**Subtype**					
Intracerebral hemorrhage	120.20 (72.21, 181.48)	3.09 (1.83, 4.69)	159.04 (93.07, 233.28)	1.97 (1.16, 2.91)	−1.55 (−1.90, −1.19)
Ischemic stroke	62.11 (32.31, 99.55)	1.81 (0.92, 2.98)	128.69 (71.55, 195.78)	1.66 (0.91, 2.55)	−0.36 (−0.52, −0.19)
Subarachnoid hemorrhage	25.11 (12.48, 40.47)	0.63 (0.31, 1.01)	17.54 (9.13, 27.82)	0.22 (0.11, 0.34)	−4.37 (−4.75, −3.99)
**SDI region**					
High SDI	14.25 (4.22, 26.35)	1.37 (0.40, 2.54)	11.31 (2.9, 22.56)	0.51 (0.12, 1.02)	−3.76 (−3.92, −3.59)
High-middle SDI	47.22 (24.93, 74.74)	4.76 (2.47, 7.61)	57.33 (29.96, 89.70)	2.87 (1.50, 4.50)	−1.96 (−2.32, −1.60)
Middle SDI	74.45 (45.92, 107.61)	8.22 (5.02, 12.01)	115.99 (69.19, 163.41)	5.33 (3.18, 7.60)	−1.48 (−1.76, −1.20)
Low-middle SDI	53.15 (35.86, 72.49)	9.85 (6.60, 13.71)	90.19 (59.26, 121.28)	7.43 (4.92, 10.1)	−1.04 (−1.25, −0.82)
Low SDI	18.26 (11.84, 26.06)	8.62 (5.58, 12.35)	30.32 (19.59, 41.93)	6.91 (4.44, 9.54)	−0.87 (−1.02, −0.71)
**GBD region**					
High-income Asia Pacific	2.88 (0.55, 5.78)	1.57 (0.29, 3.16)	2.10 (0.29, 4.87)	0.35 (0.05, 0.82)	−5.47 (−5.62, −5.33)
High-income North America	4.06 (1.52, 7.02)	1.11 (0.41, 1.93)	3.61 (0.92, 7.04)	0.50 (0.12, 0.99)	−3.03 (−3.22, −2.85)
Western Europe	9.64 (3.32, 17.27)	1.63 (0.56, 2.94)	6.88 (2.34, 13.13)	0.59 (0.19, 1.13)	−3.84 (−4.01, −3.68)
Australasia	0.41 (0.18, 0.66)	1.83 (0.83, 3.01)	0.42 (0.18, 0.72)	0.73 (0.30, 1.25)	−3.57 (−3.74, −3.40)
Southern Latin America	0.56 (0.03, 1.28)	1.26 (0.06, 2.89)	0.52 (0.08, 1.15)	0.61 (0.09, 1.35)	−2.84 (−3.01, −2.68)
Andean Latin America	0.49 (0.21, 0.77)	2.49 (1.13, 3.95)	0.68 (0.31, 1.13)	1.26 (0.59, 2.08)	−2.41 (−2.57, −2.25)
Tropical Latin America	4.07 (1.95, 6.38)	4.88 (2.37, 7.60)	4.26 (1.97, 6.86)	1.84 (0.86, 2.97)	−3.45 (−3.52, −3.38)
Central Latin America	2.78 (1.78, 3.85)	3.71 (2.37, 5.16)	4.66 (2.84, 6.81)	2.07 (1.26, 3.03)	−2.34 (−2.45, −2.23)
Caribbean	1.33 (0.81, 1.90)	5.29 (3.20, 7.57)	2.12 (1.26, 3.15)	4.08 (2.43, 6.07)	−0.78 (−0.83, −0.74)
Eastern Europe	3.07 (0.00, 9.45)	1.19 (0.00, 3.65)	3.30 (0.06, 9.48)	0.95 (0.02, 2.75)	−1.49 (−2.15, −0.83)
Central Europe	4.13 (0.94, 7.88)	3.01 (0.67, 5.73)	4.31 (1.31, 7.93)	1.91 (0.57, 3.53)	−2.06 (−2.43, −1.70)
Central Asia	1.52 (0.42, 2.75)	3.54 (1.00, 6.38)	2.25 (0.70, 4.09)	3.86 (1.31, 6.86)	0.01 (−0.57, 0.59)
North Africa and Middle East	10.46 (6.75, 14.70)	6.91 (4.37, 9.83)	16.8 (10.33, 24.11)	4.63 (2.86, 6.69)	−1.45 (−1.60, −1.31)
South Asia	48.78 (34.08, 65.37)	9.92 (6.82, 13.49)	84.24 (57.49, 111.49)	6.79 (4.66, 9.00)	−1.47 (−1.73, −1.20)
Southeast Asia	12.13 (5.33, 19.89)	5.18 (2.25, 8.42)	23.43 (10.46, 38.17)	4.36 (1.98, 7.02)	−0.41 (−0.64, −0.18)
East Asia	90.57 (57.43, 129.32)	11.98 (7.41, 17.2)	129.35 (79.91, 184.35)	7.01 (4.33, 10.01)	−1.85 (−2.19, −1.50)
Oceania	0.05 (0.00, 0.12)	1.92 (0.22, 4.65)	0.09 (0.01, 0.25)	1.73 (0.22, 4.17)	−0.36 (−0.48, −0.24)
Western Sub-Saharan Africa	3.42 (1.55, 5.61)	4.35 (1.97, 7.15)	6.05 (3.01, 9.64)	3.91 (1.97, 6.08)	−0.40 (−0.59, −0.21)
Eastern Sub-Saharan Africa	5.38 (3.20, 8.13)	8.06 (4.80, 12.02)	7.06 (3.71, 11.00)	5.44 (2.99, 8.31)	−1.46 (−1.59, −1.34)
Central Sub-Saharan Africa	1.05 (0.46, 1.74)	5.28 (2.39, 8.68)	2.03 (0.96, 3.32)	4.73 (2.38, 7.50)	−0.38 (−0.52, −0.24)
Southern Sub-Saharan Africa	0.64 (0.26, 1.09)	2.52 (1.03, 4.26)	1.13 (0.48, 1.92)	2.37 (1.01, 4.00)	−0.17 (−0.71, 0.38)

*ASMR, age-standardized mortality rate; CI, confidential interval; EAPC, estimated annual percentage change; No., number; UI, uncertainty interval.*

**TABLE 2:** Stroke DALYs and age-standardized DALYs rate attributable to lead exposure in 1990 and 2019, and its estimated annual percentage change from 1990 to 2019.

Characteristics	1990		2019		1990–2019
	DALYs No. × 10^3^ (95% UI)	ASDR per 100,000 No. (95% UI)	DALYs No. × 10^3^ (95% UI)	ASDR per 100,000 No. (95% UI)	EAPC in ASDR No. (95% CI)
Overall	5436.72 (3253.44, 7951.79)	133.36 (80.09, 195.09)	6738.78 (3912.20, 9815.60)	81.97 (47.86, 119.11)	−1.74 (−1.95, −1.52)
**Sex**					
Males	3460.84 (2171.31, 4910.60)	180.38 (113.86, 253.97)	4283.62 (2608.72, 6130.03)	111.44 (68.25, 158.73)	−1.69 (−1.93, −1.46)
Females	1975.88 (1025.52, 3095.62)	92.25 (47.93, 145.04)	2455.16 (1290.64, 3838.31)	56.15 (29.45, 88.05)	−1.83 (−2.01, −1.65)
**Subtype**					
Intracerebral hemorrhage	3270.38 (1934.84, 4853.85)	78.64 (46.74, 117.52)	3659.25 (2087.96, 5468.36)	44.03 (25.12, 65.69)	−1.98 (−2.30, −1.66)
Ischemic stroke	1425.18 (762.07, 2199.25)	37.21 (19.87, 58.27)	2601.42 (1470.90, 3919.91)	32.21 (18.17, 48.71)	−0.53 (−0.69, −0.38)
Subarachnoid hemorrhage	741.16 (361.83, 1203.25)	17.51 (8.68, 28.31)	478.11 (234.92, 776.36)	5.73 (2.80, 9.26)	−4.36 (−4.66, −4.05)
**SDI region**					
High SDI	291.95 (80.63, 558.71)	28.33 (7.76, 54.32)	191.50 (40.66, 398.85)	9.83 (1.97, 21.25)	−3.89 (−4.04, −3.75)
High-middle SDI	1158.52 (613.87, 1833.69)	107.88 (56.92, 170.56)	1128.55 (575.92, 1799.60)	55.37 (28.09, 88.61)	−2.51 (−2.85, −2.16)
Middle SDI	1996.67 (1197.41, 2904.92)	187.83 (114.35, 269.76)	2543.93 (1453.79, 3737.79)	104.78 (60.63, 152.92)	−1.98 (−2.22, −1.74)
Low-middle SDI	1475.14 (997.32, 2024.78)	232.88 (158.20, 319.29)	2106.37 (1351.01, 2888.39)	155.33 (100.91, 209.70)	−1.42 (−1.61, −1.23)
Low SDI	512.32 (326.77, 738.06)	204.43 (131.31, 293.33)	765.38 (477.87, 1079.86)	147.59 (93.99, 205.99)	−1.22 (−1.37, −1.07)
**GBD region**					
High-income Asia Pacific	64.82 (11.61, 134.74)	32.54 (5.76, 67.38)	33.58 (3.80, 81.8)	6.93 (0.66, 18.08)	−5.58 (−5.71, −5.45)
High-income North America	84.34 (28.54, 152.41)	24.08 (7.93, 43.62)	63.45 (12.18, 133.93)	9.70 (1.69, 21.12)	−3.29 (−3.43, −3.15)
Western Europe	170.53 (57.19, 308.67)	29.42 (9.67, 53.73)	91.66 (26.74, 174.41)	9.01 (2.42, 17.95)	−4.39 (−4.56, −4.22)
Australasia	7.90 (3.45, 12.98)	34.16 (14.84, 55.64)	5.95 (2.33, 10.28)	11.31 (4.19, 19.80)	−4.08 (−4.21, −3.95)
Southern Latin America	14.47 (0.73, 33.17)	31.22 (1.53, 71.46)	10.25 (1.19, 23.82)	12.35 (1.38, 28.99)	−3.61 (−3.80, −3.42)
Andean Latin America	13.42 (5.48, 22.00)	60.51 (25.99, 97.34)	14.83 (6.03, 25.99)	26.34 (10.91, 45.49)	−2.96 (−3.12, −2.81)
Tropical Latin America	109.82 (48.40, 176.91)	113.85 (52.47, 180.67)	86.93 (35.98, 147.39)	36.12 (15.11, 60.63)	−4.09 (−4.19, −4.00)
Central Latin America	70.30 (43.04, 99.91)	81.37 (51.20, 113.30)	94.1 (54.11, 140.78)	40.23 (23.44, 59.48)	−2.75 (−2.86, −2.64)
Caribbean	33.95 (20.45, 48.80)	128.17 (77.57, 183.87)	45.89 (26.00, 70.13)	88.56 (50.07, 135.55)	−1.18 (−1.23, −1.13)
Eastern Europe	68.54 (0.07, 212.13)	24.76 (0.03, 76.72)	66.06 (0.97, 194.76)	19.29 (0.27, 57.51)	−1.57 (−2.29, −0.85)
Central Europe	94.01 (21.60, 180.31)	64.39 (14.43, 123.79)	75.28 (20.80, 142.85)	34.50 (9.11, 66.57)	−2.68 (−3.06, −2.29)
Central Asia	36.50 (9.22, 68.35)	78.04 (20.18, 144.12)	51.65 (13.51, 99.21)	74.17 (22.01, 137.55)	−0.47 (−1.02, 0.07)
North Africa and Middle East	290.08 (184.52, 408.97)	161.25 (103.86, 224.46)	406.62 (242.62, 592.39)	95.53 (57.79, 137.74)	−1.88 (−2.03, −1.73)
South Asia	1360.34 (949.57, 1830.30)	227.73 (159.39, 305.32)	2013.57 (1342.51, 2720.83)	143.45 (96.95, 190.85)	−1.67 (−1.88, −1.47)
Southeast Asia	351.90 (148.41, 581.07)	128.19 (55.38, 210.75)	571.62 (231.08, 985.34)	93.72 (39.90, 158.29)	−0.91 (−1.13, −0.69)
East Asia	2376.42 (1479.43, 3330.24)	266.03 (168.14, 376.35)	2703.81 (1624.42, 3888.66)	132.64 (80.63, 190.15)	−2.40 (−2.70, −2.10)
Oceania	1.29 (0.09, 3.69)	42.69 (3.79, 113.10)	2.44 (0.15, 7.32)	35.72 (3.14, 96.48)	−0.59 (−0.72, −0.47)
Western Sub-Saharan Africa	93.48 (41.43, 154.05)	101.66 (46.09, 166.13)	152.86 (69.93, 254.15)	81.28 (39.77, 130.18)	−0.82 (−1.02, −0.62)
Eastern Sub-Saharan Africa	146.65 (82.38, 223.67)	187.67 (110.73, 281.98)	168.33 (81.62, 273.83)	108.47 (56.11, 171.59)	−2.02 (−2.15, −1.89)
Central Sub-Saharan Africa	30.44 (12.66, 51.31)	125.42 (54.78, 207.35)	53.12 (23.02, 90.09)	100.74 (46.98, 164.36)	−0.76 (−0.90, −0.61)
Southern Sub-Saharan Africa	17.55 (6.82, 30.75)	60.80 (24.38, 104.80)	26.79 (10.30, 46.83)	48.64 (19.44, 84.37)	−0.72 (−1.25, −0.19)

*ASDR, age-standardized DALYs rate; CI, confidential interval; DALYs, disability-adjusted life years; EAPC, estimated annual percentage change; No., number; UI, uncertainty interval.*

**FIGURE 1:**
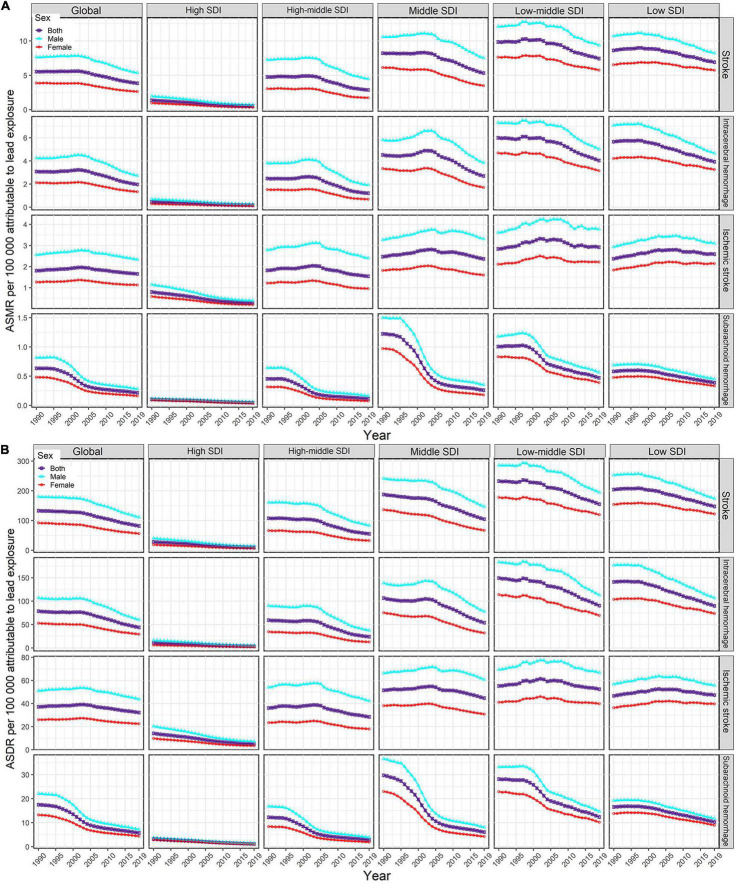
Temporal trends in age-standardized rates of lead exposure-related stroke mortality **(A)** and DALYs **(B)** from1990 to 2019 by sex, SDI region, and pathological subtype. ASMR, age-standardized mortality rate; ASDR, age-standardized DALYs rate; DALYs, disability-adjusted life years; SDI, socio-demographic index.

For the SDI regions, in 2019, the highest numbers of lead exposure-related stroke deaths and DALYs were seen in the middle-SDI region; while the highest ASMR and ASDR were observed in the low-middle-SDI region (Tables 1, 2). Both numbers and age-standardized rates of lead exposure-related stroke deaths and DALYs were lowest in the high-SDI region (Tables 1, 2 and Figure 1).

For the GBD regions, in 2019, the highest numbers of lead exposure-related stroke deaths and DALYs were seen in East Asia, while the lowest numbers of these indicators were observed in Oceania (Tables 1, 2). Meanwhile, the highest ASMR and ASDR of stroke attributable to lead exposure were observed in East Asia and South Asia, respectively (Tables 1, 2). The High-income Asia Pacific, High-income North America, and Western Europe had the lowest ASMR and ASDR (Tables 1, 2).

**FIGURE 2:**
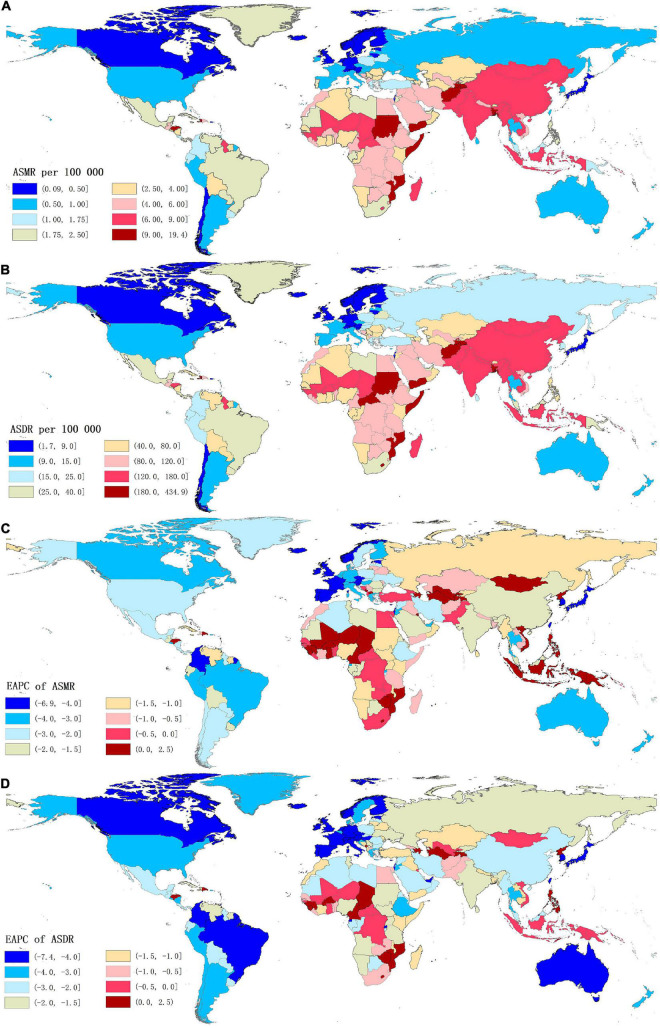
The global distribution of age-standardized rates of stroke mortality **(A)** and DALYs **(B)** attributable to lead exposure for both sexes in 204 countries in 2019, and the corresponding EAPCs of age-standardized rates of stroke mortality **(C)** and DALYs **(D)** from 1990 to 2019. ASMR, age-standardized mortality rate; ASDR, age-standardized DALYs rate; DALYs, disability-adjusted life years; EAPC, estimated annual percentage change.

For the countries, in 2019, the highest numbers of stroke deaths and DALYs attributable to lead exposure were observed in China, followed by India, Bangladesh, and Indonesia; while the lowest numbers of these indicators were seen in Niue (Supplementary Tables 1, 2). Meanwhile, Afghanistan was the country with the highest ASMR of stroke, followed by Yemen, Haiti, Bangladesh, Sudan, and Mozambique, all of which had an ASMR of more than 10.00 per 100,000 (Supplementary Table 1 and Figure 2A). The highest ASDR of lead exposure-related stroke was observed in Afghanistan while the lowest ASDR was seen in Finland (Supplementary Table 2 and Figure 2B).

### Temporal Trends of Stroke Burden Attributable to Lead Exposure From 1990 to 2019

**FIGURE 3:**
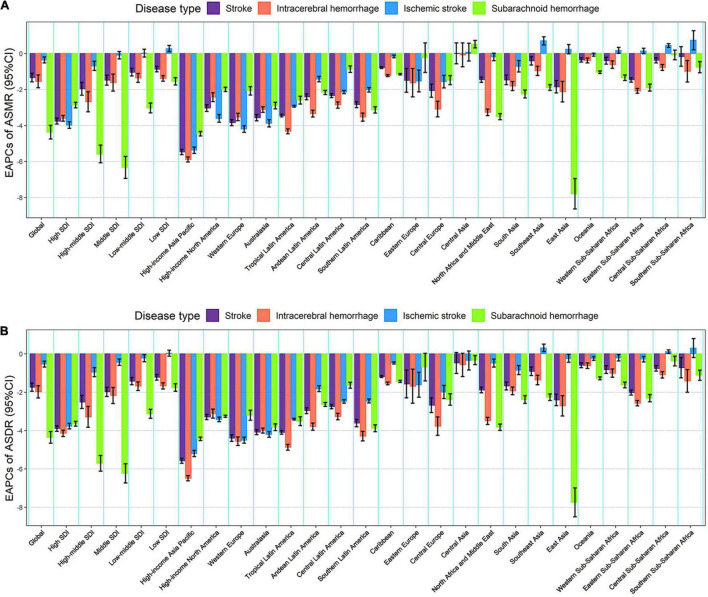
The EAPCs in age-standardized rates of lead exposure-related stroke mortality **(A)** and DALYs **(B)** from 1990 to 2019 for both sexes by SDI region, GBD region and pathological subtype. ASMR, age-standardized mortality rate; ASDR, age-standardized DALYs rate; DALYs, disability-adjusted life years; EAPC, estimated annual percentage change; SDI, socio-demographic index.

From 1990 to 2019, the global numbers of lead exposure-related stroke deaths and DALYs increased by 1.47 and 1.24 times, respectively (Tables 1, 2). However, the ASMR and ASDR decreased by 30.38 and 38.53%, and the EAPCs were −1.34 [95% CI: (−1.57, −1.10)] and −1.74 [95% CI: (−1.95, −1.52)], respectively (Tables 1, 2 and Figure 3). During the study period, both ASMR and ASDR of stroke attributable to lead exposure were decreased in all SDI regions, with the highest decrease in the high-SDI region [ASMR: EAPC = −3.76, 95% CI: (−3.92, −3.59); ASDR: EAPC = −3.89, 95% CI: (−4.04, −3.75)] (Tables 1, 2 and Figure 3).

At the GBD regional level, the ASMR and ASDR of lead exposure-related stroke decreased in most GBD regions, especially in High-income Asia Pacific [ASMR: EAPC = −5.47, 95% CI: (−5.62, −5.33); ASDR: EAPC = −5.58, 95% CI: (−5.71, −5.45)] (Tables 1, 2 and Figure 3). However, the ASMR and ASDR remained stable in Central Asia (Tables 1, 2 and Figure 3). At the national level, downward trends of ASMR and ASDR of lead exposure-related stroke were observed in 80.88 and 89.22% of the countries, respectively, and South Korea was the country with the highest decrease [ASMR: EAPC = −6.85, 95% CI: (−7.15, −6.56); ASDR: EAPC = −7.35, 95% CI: (−7.61, −7.09)] (Supplementary Tables 1, 2 and Figures 2C,D). However, the highest upward trends in ASMR and ASDR were seen in the Philippines [ASMR: EAPC = 2.44, 95% CI: (1.74, 3.16); ASDR: EAPC = 2.47, 95% CI: (1.74, 3.19)] (Supplementary Tables 1, 2 and Figures 2C,D).

### The Global Burden and Temporal Trends of Stroke Attributable to Lead Exposure by Pathological Subtypes

For pathological subtypes, in 2019, more than half of global lead exposure-related stroke deaths and DALYs occurred in ICH, followed by IS and SAH (Tables 1, 2, Figure 1, and Supplementary Figure 1). In 2019, ICH occupied the highest proportions of ASMR and ASDR in the low to middle SDI regions, while IS was the leading contributor to the ASMR and ASDR in the high-middle and high SDI regions (Figure 1 and Supplementary Figure 1). IS accounted for higher proportions of ASMR in most GBD regions and countries in 2019 (11 out of 21 GBD regions and 110 out of 204 countries), followed by ICH (10 out of 21 GBD regions and 94 out of 204 countries). However, for the ASDR, ICH was the leading contributor in most GBD regions and countries in 2019 (13 out of 21 GBD regions and 108 out of 204 countries).

From 1990 to 2019, the global numbers of lead exposure-related stroke deaths and DALYs increased in ICH and IS while decreasing in SAH (Tables 1, 2). During this study period, downward trends of global ASMR and ASDR of stroke attributable to lead exposure were observed in all pathological subtypes (Tables 1, 2). Meanwhile, from 1990 to 2019, the ASMR and ASDR of ICH and SAH decreased in all SDI regions and GBD regions (except for Central Asia), while the ASMR and ASDR of IS decreased in most SDI regions and GBD regions (Figure 3).

### The Global Burden and Temporal Trends of Stroke Attributable to Lead Exposure by Age and Sex

Both the global numbers and age-standardized rates of mortality and DALYs of lead exposure-related stroke were higher in males than those in females in 2019 (Tables 1, 2). From 1990 to 2019, in both sexes, the numbers of deaths and DALYs of stroke attributable to lead exposure increased, while the ASMR and ASDR decreased, and the decline in EAPC in females was more pronounced than in males (Tables 1, 2 and Figure 1).

**FIGURE 4:**
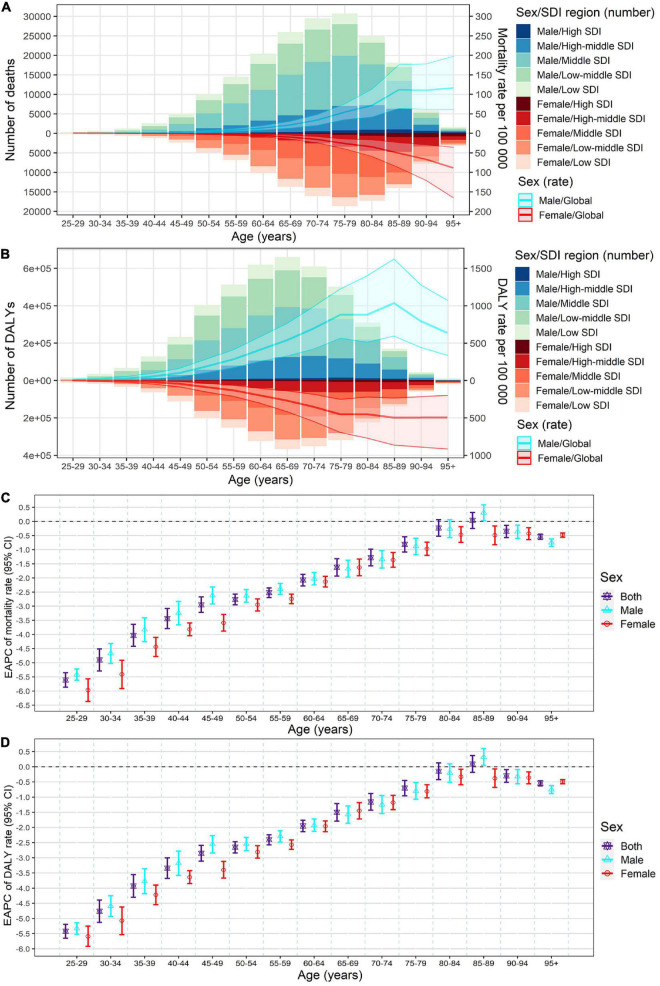
Age-specific numbers and rates of stroke mortality **(A)** and DALYs **(B)** attributable to lead exposure by sex and SDI region in 2019, and the corresponding EAPCs of age-specific rates of mortality **(C)** and DALYs **(D)** by sex from1990 to 2019. The bar plots represented the numbers of deaths and DALYs; the line plots and their shade represented the rates mortality and DALYs and their 95%UI. DALYs, disability-adjusted life years; EAPC, estimated annual percentage change; SDI, socio-demographic index.

Meanwhile, in both sexes, the global numbers of lead exposure-related stroke deaths peaked in the 75–79 age group, while the numbers of DALYs peaked in the 65–69 age group in 2019 (Figures 4A,B). The global age-specific rates of mortality and DALYs increased with age, however, the age-specific DALYs rate decreased after 85 years old in both sexes in 2019 (Figures 4A,B). From 1990 to 2019, downward trends of both age-specific rates of mortality and DALYs were observed in most age groups of both sexes, except for the 80–89 age groups (Figures 4C,D).

### The Influential Factors for Estimated Annual Percentage Change

At the national level, a significant positive association was observed between Estimated Annual Percentage Change (EAPC) and lead exposure-related stroke burden rates (ASMR and ASDR) in 1990 (ρ = 0.3974, *P* < 4.0e−09 for ASMR; ρ = 0.3911, *P* < 7.3e−09 for ASDR) (Supplementary Figures 2A,B). Meanwhile, we also observed that there was a significant negative association between EAPC and SDI in 2019 at the national level (ρ = −0.6535, *P* < 2.2e−16 for ASMR; ρ = −0.6490, *P* < 2.2e−16 for ASDR) (Supplementary Figures 2C,D). Moreover, we assessed the association between lead exposure-related stroke burden rates (ASMR and ASDR) and SDI from 1990 to 2019 at the GBD region level and pronounced negative associations were observed (Supplementary Figure 3).

## Discussion

Although the burden of stroke has declined during the past few decades, stroke remains an important health problem in the world (2, 3). The cost of stroke care brings a high burden on both society and patients’ families (13), and the first primary prevention of stroke is the most effective way to reduce the burden (9). Lead is an important environmental pollutant, and lead exposure caused nearly 5% of the total number of stroke deaths and DALYs in 2019 (8). In low and middle-income countries, the proportion of stroke burden attributable to lead exposure was higher (8). Fully assessing the burden of stroke attributable to lead exposure is essential for the effective prevention of stroke. Therefore, using the GBD database, we comprehensively evaluated the burden of stroke attributable to lead exposure by age, sex, pathological subtype, region, and country.

We found that, in 2019, the highest burden of stroke attributable to lead exposure was seen in males, the low and low-middle SDI regions, and the ICH subtype, which was consistent with the overall trend of stroke (2, 3). Meanwhile, from 1990 to 2019, the global numbers of stroke deaths and DALYs attributable to lead exposure increased, which could be attributed to the growth of the population and aging (2, 3). Moreover, the ASMR and ASDR decreased during the study period, among which females, the high SDI region, and the SAH subtype declined most significantly; and accordingly, downward trends in ASMR and ASDR were also seen in most GBD regions and countries.

In this present study, the changing trends of stroke ASMR and ASDR attributable to lead exposure were different between countries and regions. The implementation of primary strategies to reduce lead exposure was an important reason for the decline. In the 1970s, high-income countries implemented a policy to ban the use of lead in fuels and paints, resulting in a significant decline in the blood level of lead in the population (32–35). However, in low and middle-income countries, this problem had been resolved late (34). In the “toxic hotspots” (where lead-acid battery recycling, lead mining and smelting, and electronics recycling were conducted without environmental protections) in low and middle-income countries, lead exposure might cause important local health problems (36). Meanwhile, socio-economic development is another important reason. From our study, we mainly observed a significant positive association between EAPC and stroke burden rates, and a significant negative association between EAPC and SDI, which indicated that the lead exposure-related stroke burden might be given priority intervention in countries with high socio-economic development. Countries with high socio-economic development often have better medical and health care conditions and more effective implementation of prevention strategies (2). Accordingly, the improved high-efficiency treatment, the improvement of stroke acute health care, and the improvement of people’s awareness of stroke and the hazards of lead exposure might also contribute to the global decline of stroke burden related to lead exposure (2, 3).

The above factors would lead to two effects. On the one hand, the improved medical care and treatment could significantly improve survival, which could lead to a reduction in mortality and DALYs of stroke attributable to lead exposure (especially in high-income countries and regions); and on the other hand, due to unbalanced development and differences in medical conditions, the changing trend of stroke burden attributable to lead exposure varied between countries and regions, which highlights the urgency of taking preventive strategies in low and middle-income countries. Therefore, for the low and middle-income countries, the burden of lead exposure-related stroke could be reduced by the following methods: firstly, to control lead exposure by optimizing the industrial processes, strengthening environmental monitoring, and improving the living environment; secondly, to improve quality of survival by raising awareness of the whole population and developing the medical technology.

The stroke burden attributable to lead exposure also showed sex differences. It could be observed that the lead exposure-related stroke burden was higher in males than females over the past 30 years. Many reasons can explain the above difference. Firstly, compared with females, males have a higher proportion of occupations related to lead exposure (such as construction and mechanical workers), which increases their chances of occupational exposure (37). Second, males have more exposure to stroke-related risk factors, such as smoking and alcohol drinking (26), which may interact with lead exposure to affect the development of stroke. Blood lead levels are associated with smoking and alcohol consumption (10, 38), which increases the level of male lead exposure. Notably, due to the cumulative effect (9), blood lead levels are significantly increased with age (10), which would result in age differences. Consistent with this hypothesis, in our study, we found that the age-specific mortality and DALYs rates of stroke attributed to lead exposure increased with age in 2019.

In addition, we further evaluated the burden of lead exposure-related stroke by pathological subtypes and found that more than half of the global stroke burden attributed to lead exposure occurred in ICH in 2019, however, the proportions of pathological types varied with socio-economic development. It could be seen that ICH occupied the highest proportions in the low to middle SDI regions, while IS was the leading contributor in the high-middle and high SDI regions. Furthermore, from 1990 to 2019, the burden of ICH and SAH subtypes of stroke (attributed to lead exposure) decreased in all SDI regions and GBD regions (except for Central Asia), while the burden of IS subtype increased in some regions. The pathological subtype difference in stroke burden might be related to socio-economic development and the interaction between lead exposure and other risk factors, which is worthy of further study (2, 5).

### Study Limitations

There were some limitations in our study, which should be taken into account in the interpretation of the results. Firstly, although the GBD study (2019) collected data systematically and comprehensively, data in some countries, especially in low and middle-income countries, were still less available. Secondly, the GBD study used a variety of statistical models to analyze the data, however, bias could not be avoided in fitting the data. Thirdly, some types of stroke (such as silent stroke) were not included in the GBD data, this might lead to an underestimation of the stroke burden. Lastly, we should not neglect that the proportion of the population included in the GBD study might have changed over the past 30 years, therefore, it should be fully considered when interpreting the results.

## Conclusion

Our study demonstrated that the stroke burden attributable to lead exposure was still high in males, the elderly population, low and middle-income countries, and the ICH subtype in 2019. However, the ASMR and ASDR decreased from 1990 to 2019 in most regions and countries, especially in females, the high SDI region, and the SAH subtype. To reduce the stroke burden attributable to lead exposure, it is important to continue to implement primary prevention strategies for lead exposure, especially in low and middle-income countries and regions. Meanwhile, high-efficiency treatment and stroke acute health care should also be continuously improved.

## Data Availability Statement

Publicly available datasets were analyzed in this study. This data can be found here: http://ghdx.healthdata.org/gbd-results-tool.

## Ethics Statement

The studies involving human participants were reviewed and approved by the Institutional Review Boards of Qilu Hospital of Shandong University with approval number KYLL-202011(KS)-239. Written informed consent for participation was not required for this study in accordance with the national legislation and the institutional requirements.

## Author Contributions

TZ, XY, and ML: conceptualization. TZ, XY, YZ, HC, JM, YL, and JC: data curation, methodology, formal analysis, software, and visualization. TZ: writing – original draft. XY and ML: funding acquisition and writing – review and editing. All authors approved the final manuscript for submission.

## Conflict of Interest

The authors declare that the research was conducted in the absence of any commercial or financial relationships that could be construed as a potential conflict of interest.

## Publisher’s Note

All claims expressed in this article are solely those of the authors and do not necessarily represent those of their affiliated organizations, or those of the publisher, the editors and the reviewers. Any product that may be evaluated in this article, or claim that may be made by its manufacturer, is not guaranteed or endorsed by the publisher.

## Footnotes

1
http://ghdx.healthdata.org/gbd-results-tool

## Supplementary Material

Supplementary Figure 1 Proportions of ischemic stroke, intracerebral hemorrhage, and subarachnoid hemorrhage of total number of lead exposure-related stroke deaths **(A)** and DALYs **(B)**, both sexes, by SDI and GBD region, in 1990 and 2019. DALYs, disability-adjusted life years; SDI, socio-demographic index.

Supplementary Figure 2 The influence factors of the EAPCs in age-standardized rates of lead exposure-related stroke mortality and DALYs for both sexes in 204 countries from 1990 to 2019. **(A)** ASMR of stroke in 1990 and EAPC in ASMR; **(B)** ASDR of stroke in 1990 and EAPC in ASDR; **(C)** socio-demographic index in 2019 and EAPC in ASMR; **(D)** socio-demographic index in 2019 and EAPC in ASDR. The circle represented the country, and the size of the circle represented the number of deaths or DALYs. The ρ indices and *P*-values were evaluated by Spearman rank analysis. The blue line and its shade were fitted by LOESS. ASMR, age-standardized mortality rate; ASDR, age-standardized DALYs rate; DALYs, disability-adjusted life years; EAPC, estimated annual percentage change; SDI, socio-demographic index.

Supplementary Figure 3 The changing trend in age-standardized rates of lead exposure-related stroke mortality **(A)** and DALYs **(B)** across GBD regions with socio-demographic index, both sexes, from 1990 to 2019. The blue line and its shade were fitted by LOESS. ASMR, age-standardized mortality rate; ASDR, age-standardized DALYs rate; DALYs, disability-adjusted life years; EAPC, estimated annual percentage change.

## Abbreviations

ASDR: age-standardized DALYs rate
ASMR: age-standardized mortality rate
CI: confidence interval
DALYs: disability-adjusted life years
EAPC: estimated annual percentage change
GBD: The Global Burden of Diseases Injuries and Risk Factors Study
ICH: intracerebral hemorrhage
IS: ischemic stroke
SAH: subarachnoid hemorrhage
SDI: socio-demographic index
UI: uncertainty interval
WHO: World Health Organization.
