# Supplementary figures and images for: Global Trends in Mortality and Burden of Stroke Attributable to Lead Exposure From 1990 to 2019
Source: Front Cardiovasc Med. 2022 Jun 23;9:870747. doi: 10.3389/fcvm.2022.870747 (PMC9259800; doi:10.3389/fcvm.2022.870747)

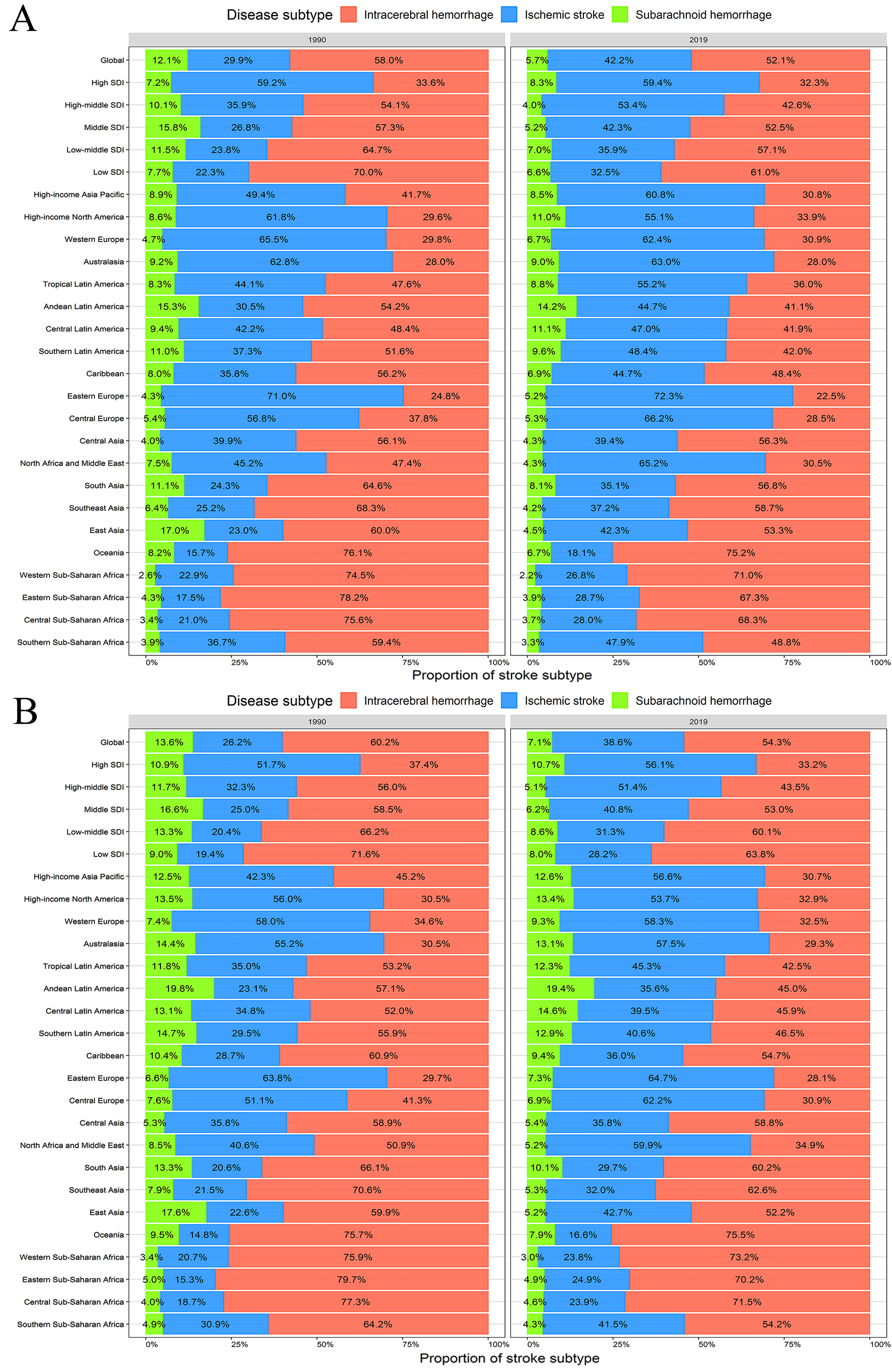

Supplement: Supplementary Figure 1 — Proportions of ischemic stroke, intracerebral hemorrhage, and subarachnoid hemorrhage of total number of lead exposure-related stroke deaths (A) and DALYs (B), both sexes, by SDI and GBD region, in 1990 and 2019. DALYs, disability-adjusted life years; SDI, socio-demographic index. [file Image_1.JPEG]

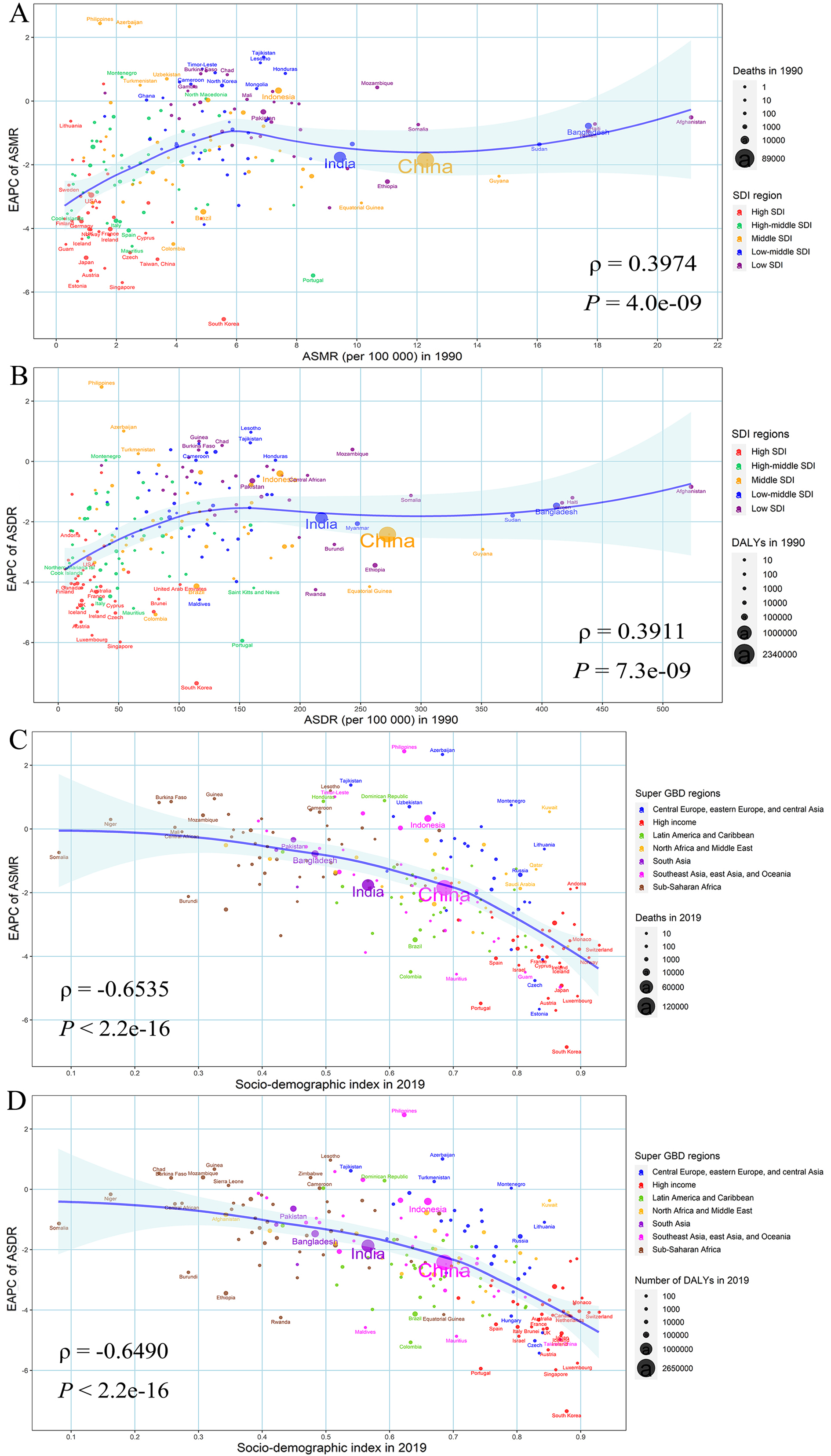

Supplement: Supplementary Figure 2 — The influence factors of the EAPCs in age-standardized rates of lead exposure-related stroke mortality and DALYs for both sexes in 204 countries from 1990 to 2019. (A) ASMR of stroke in 1990 and EAPC in ASMR; (B) ASDR of stroke in 1990 and EAPC in ASDR; (C) socio-demographic index in 2019 and EAPC in ASMR; (D) socio-demographic index in 2019 and EAPC in ASDR. The circle represented the country, and the size of the circle represented the number of deaths or DALYs. The ρ indices and P-values were evaluated by Spearman rank analysis. The blue line and its shade were fitted by LOESS. ASMR, age-standardized mortality rate; ASDR, age-standardized DALYs rate; DALYs, disability-adjusted life years; EAPC, estimated annual percentage change; SDI, socio-demographic index. [file Image_2.JPEG]

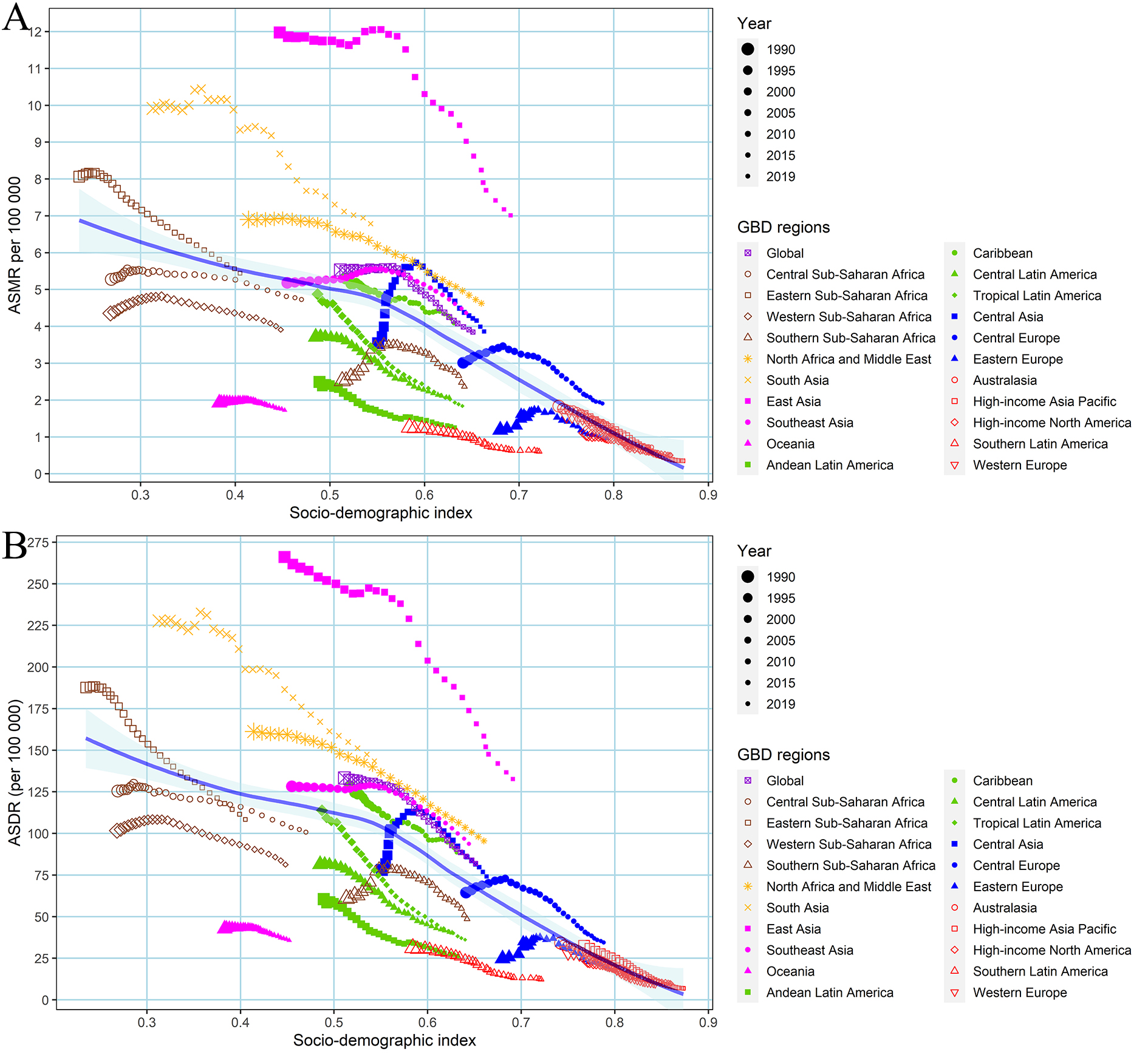

Supplement: Supplementary Figure 3 — The changing trend in age-standardized rates of lead exposure-related stroke mortality (A) and DALYs (B) across GBD regions with socio-demographic index, both sexes, from 1990 to 2019. The blue line and its shade were fitted by LOESS. ASMR, age-standardized mortality rate; ASDR, age-standardized DALYs rate; DALYs, disability-adjusted life years; EAPC, estimated annual percentage change. [file Image_3.JPEG]
